# Central plasticity and dysfunction elicited by aural deprivation in the critical period

**DOI:** 10.3389/fncir.2015.00026

**Published:** 2015-06-02

**Authors:** Zhiji Chen, Wei Yuan

**Affiliations:** Department of Otorhinolaryngology Head and Neck Surgery, Southwest Hospital, Third Military Medical UniversityChongqing, China

**Keywords:** sensory deprivation, auditory cortex, neuronal plasticity, critical period, auditory perceptual disorders, correction of hearing impairment

## Abstract

The acoustic signal is crucial for animals to obtain information from the surrounding environment. Like other sensory modalities, the central auditory system undergoes adaptive changes (i.e., plasticity) during the developmental stage as well as other stages of life. Owing to its plasticity, auditory centers may be susceptible to various factors, such as medical intervention, variation in ambient acoustic signals and lesion of the peripheral hearing organ. There are critical periods during which auditory centers are vulnerable to abnormal experiences. Particularly in the early postnatal development period, aural inputs are essential for functional maturity of auditory centers. An aural deprivation model, which can be achieved by attenuating or blocking the peripheral acoustic afferent input to the auditory center, is ideal for investigating plastic changes of auditory centers. Generally, auditory plasticity includes structural and functional changes, some of which can be irreversible. Aural deprivation can distort tonotopic maps, disrupt the binaural integration, reorganize the neural network and change the synaptic transmission in the primary auditory cortex or at lower levels of the auditory system. The regulation of specific gene expression and the modified signal pathway may be the deep molecular mechanism of these plastic changes. By studying this model, researchers may explore the pathogenesis of hearing loss and reveal plastic changes of the auditory cortex, facilitating the therapeutic advancement in patients with severe hearing loss. After summarizing developmental features of auditory centers in auditory deprived animals and discussing changes of central auditory remodeling in hearing loss patients, we aim at stressing the significant of an early and well-designed auditory training program for the hearing rehabilitation.

The sense of hearing is a major pathway by which humans use to recognize environment. Acoustic cues assist humans to engage in neural activities integrated from multiple brain stations, which make constant adjustment of their behavior, such as learning, communication and exercise. Hearing impairment directly affects the recognition of language and social competency among humans. Hearing relies on the signal transmission from the auditory organ to the central auditory system. The central auditory system consists of cochlear nucleus, superior olive nucleus, lateral lemniscus, inferior colliculus, medial geniculation body and auditory cortex. Earlier studies suggested connections between different stations of the auditory system which are structurally hard-wired. This view was however challenged by recent research upon discovering the plasticity of sensory systems (including the auditory system; Syka, 2002; Sanes and Bao, 2009; Kral, 2013). Both clinical and basic researches suggest that plastic changes of the auditory system can take place throughout the lifetime of humans and animals.

Plasticity is both structural and functional reorganization of the brain to adapt to ambient changes and physiological changes in the body (Jain et al., 1998; Rittenhouse et al., 1999; Pascual-Leone et al., 2005). Factors such as life experiences, afferent signals, electrical stimulations and the learning of new techniques are considered to be important for regulating brain function and remodeling neuronal circuits (May, 2011). Central auditory plasticity is observed not only during childhood but also in some adults with congenital deafness who benefit from cochlear implants, confirming that the dysfunctional auditory system can still undergo plastic changes (Syka, 2002; Berrettini et al., 2011). Studies on central auditory plasticity have greatly expanded theories presented in the field of audiology. Meanwhile, these discoveries have motivated the audiologists to better auditory interventions. In this review, we make an elucidation with the “critical period” and summarize research findings of the auditory deprived model to discuss the connection between the auditory plasticity and the rehabilitation strategy.

## Critical Period of Auditory Center During Developmental Stage

The plasticity of auditory center does not always stay the same throughout the lifetime. There are sensitive periods when the auditory center is more susceptible to ambient environment and audio input (Takesian et al., 2009; Kral, 2013). In these periods, brain can reorganize with structural and functional changes. For humans, the sound localization, the frequency discrimination, the tonotopicity and the cochleotopic map, can all be affected. Critical period is suggested as a period time during which some plastic changes of the auditory system may occur irreversibly and cannot be compensated later in life (Hensch, 2004). But there are multiple developmental windows during which the functional organization of sensory brain regions can be affected by sensory experience, and experience-dependent plasticity does not exert its effect only in one critical period. By training adult animals, auditory feature representation of the primary auditory cortex can be changed (Polley et al., 2006). The end of the critical period is more like the developmental transition between exposure-based plasticity and reinforcement-based plasticity (Popescu and Polley, 2010).

Some researchers revealed the time limit of critical period by studying developmental and functional characteristics of brain. The external auditory canal opened on postnatal day (P) 9 in rats, and detection of the auditory brainstem response (ABR) on P12 demonstrated the auditory function. The hearing threshold detected on P22 was close to that in adulthood (Geal-Dor et al., 1993). This time period is considered to be “critical” for the hearing development of rats, in which the plasticity of auditory cortex is significantly higher than in other periods (Kral, 2013). Moreover it is also known as an important process for individual adaptation to the environment (Hensch, 2004; Sanes and Bao, 2009). For example, neonatal rats exposed to a pure tone indeed exhibited an expanded region of the primary auditory cortex corresponding to characteristic frequency, and this change could be sustained into adulthood; in contrast, mothers of these rats showed no changes in the same acoustic environment (Zhang et al., 2001). In addition to this, a study by Zhang et al. showed that postnatal 9–28 days (P9–P28) rats exposed to the pulsed white noise displayed disrupted tonotopicity and impaired frequency-response selectivity in the neurons of their primary auditory cortex in adulthood, and also a number of selective changes were found in the primary auditory cortex of neonatal rats when exposed to a complex tone sequence (Zhang et al., 2002; Nakahara et al., 2004). Furthermore, Merzenich’s study suggested that pure tone exposure from postnatal day 11 (P11) to P13 affected the sound representation and the cortical representation of sound intensity (de Villers-Sidani et al., 2007). However, noise exposure after P30 resulted in no significant damage in the cortical auditory map of the rats, suggesting that the pattern of auditory input during critical period plays an irreplaceable role in shaping the decoding circuit of the primary auditory cortex (Zhang et al., 2002; Nakahara et al., 2004). This line of research confirmed that the acoustic environment must be essential for the auditory cortex development of neonatal rats during the first postnatal month.

The impact of impaired input on the central nervous system involve various processes, however only some of these changes are reversible when signal inputs recover before the end of the critical period. In a recent study, Mowery et al. (2014) used whole cell patch clamp recording in brain slices after inserting earplugs to cut off air borne sound in the critical period, and they revealed that: (1) the characteristic of cytomembrane, the action potential and the inhibitory postsynaptic current were susceptible to the deafferentation (even in a transient time) during the critical period; (2) early regained input of the auditory signal before the end of the critical period resulted in a better functional recovery of the cells, even though the discharge frequency sustained damage; (3) if the blockade of signal input was kept beyond the critical period, some of changes would probably continue into adulthood.

Undoubtedly, clinical researchers believe that a transient blockade of the auditory afferentiation in the critical period may result in a chronic impairment of brain function, delaying the acquisition of the auditory skills in deaf children. After comparison and meta-analyses of the literature, Whitton showed that infants with severe otitis media displaying incoming signal attenuation were at a high risk of developing into a persistent central auditory impairment (Whitton and Polley, 2011). These infants were under a condition called “amblyaudio”, much like the “amblyopia”. According to the research on the monaural deprivation, the mechanism was multifaceted, including binaural integration, auditory reorganization, and maladaptive plasticity during critical periods of auditory cortex development (Popescu and Polley, 2010). With the critical period as a core, Kral reviewed the early developmental features of auditory center to evaluate the significance of auditory experience in functional maturation and to integrate results of his studies on the postoperative development of the nerve center after cochlear implantation (Kral et al., 2006; Kral and Sharma, 2012; Kral, 2013). He suggested that at least five mechanisms associated with neural signal processing and central development could affect auditory recovery in animals and humans, proposing that molecular intervention in the future may alter the reorganization of auditory center caused by sensory experience (Kral, 2013).

## Establishment of Aural Deprivation Models

Since the critical period is so important, it is necessary to set up a model to figure out the underlying mechanisms of auditory plasticity. Clinical findings showed that if patients suffering from bilateral symmetrical hearing impairment only wore one hearing aid, their speech recognition score in the ear without hearing aid might subsequently decline, whereas the pure tone hearing threshold and speech recognition threshold showed no change. This phenomenon was referred to as the late-onset aural deprivation (Silman et al., 1984). Over a decade later, many laboratories have reported their extensive findings in this confused area. In 1996, Eriksholm and colleagues summarized their findings and proposed the concept of “aural deprivation effect”, indicating that a reduction of acoustic information leads to a gradual decline in the auditory function. They determined the association between the acquisition of sensory signals and the development of the central nervous system (Arlinger et al., 1996). Similar to the development of visual system, attenuation or bilateral signal input imbalances may also lead to changes in the plasticity of auditory center, resulting in a chronic hearing impairment.

For studying the plasticity of auditory center, establishing an *in vivo* model involving aural deprivation is a commonly employed technique. In order to establish this model, previous studies have applied different strategies by far, including the use of medication, noise, cochlear ablation, earplug insertion and a silent environment, to eliminate incoming acoustic signals. Among these models, complete or partial cochlear ablation has been the most conventional method employed to study plastic changes of the center after aural deprivation. This method aims to terminate the peripheral signal to stimulate the auditory center through ascending transduction pathway, which has been proved more stable than the drug-induced aural deprivation in establishing *in vivo* models, and it also avoids drug-induced damage to the vestibular function (Heydt et al., 2004; de Groot et al., 2005). In addition, by collecting data from the same animal, this model can be used to perform side-by-side comparisons between impaired and untreated ears. Presbycusis and noise-induced deafness also show damages in the peripheral hearing organ, thereby further affecting the auditory signal input. However, these models may also be influenced by degeneration and damage in the auditory center, resulting in a relatively complicated injury mechanism. About the operative procedure of cochlea ablation, Mostafapour successfully established a mouse model involving cochlear resection from the tympanic membrane in 2000 to assess cellular apoptosis of the cochlear nucleus after a surgery (Mostafapour et al., 2000). Subsequently, Zhang et al. (2009) applied an innovative approach to remove the cochlea in an opisthotic pathway. In addition, the earplug insertion can be employed to compel neonatal rats in sound shield. A study by Wang demonstrated that this air-conduction aural deprivation increased the auditory threshold and reduced the amplitude of the ABR in these rats; most importantly, auditory nucleus of these animals also showed apoptosis of type I spiral ganglion neurons and other cells (Wang et al., 2011). By partial spiral ganglion lesions, Snyder established an aural deprivation model with a cat, in which inferior colliculus neurons could tune to the neighboring frequencies (the lesion edge frequencies) more sensitively, but less to the characteristic frequencies (the lesion frequencies; Snyder et al., 2000; Snyder and Sinex, 2002). Besides, shaker-2 mice and congenital deaf cats with genetic cochlea damage can also be used to study the plasticity (Kral et al., 2002; Lee et al., 2003a; Tirko and Ryugo, 2012).

## Morphological and Functional Changes in the Central Nervous System Following Aural Deprivation

Aural deprivation will lead to numerous changes in the central nervous system, including a decreasing volume and number of neuron in the spiral ganglion as well as in the central auditory nucleus, the reconstruction of neural projections, the regulation of intracortical and thalamocortical neural activities, the dendrite pruning in pyramidal cells and so on. As listed above, these all can delay the maturity of auditory center, reorganize the neural network and reduce the effect of the cochlear implantation. In particular, aural deprivation may significantly up-regulate neuronal apoptosis of the nucleus in ascending auditory pathway. Early studies (for example, employing cochlear ablation, ear bone resection or tetrodotoxin blockade of acoustic nerve activity as *in vivo* models for aural deprivation) showed a reduced volume of the cochlear nucleus after aural deprivation. Later studies revealed that the degree of apoptosis after aural deprivation was associated with the developmental stage of the affected mice. A comparison of aural deprivation in P5–7 and P21 mice showed that the P5–7 mice displayed earlier neuronal apoptosis in the cochlear nucleus than the P21 mice (Kim et al., 1997). Establishing a mouse model with bilateral cochlear resection, researchers quantified the reduced volume of cochlear nucleus and the neuronal loss in different regions of cochlea. The results revealed a significant change in the number of octopus cells in posteroventral cochlear nucleus (Zhang et al., 2009). According to Mostafapour’s research, cochlea resection of wild type mouse would lead to 61% neuron loss in anterior ventral cochlea nucleus (ACVN) on postnatal 5 days (P5), but less than 1% when resection on P14 (Mostafapour et al., 2000). Apart from neuronal loss in the spiral ganglion and the cochlear nucleus, it had also been observed in the medial geniculate body and the auditory cortex 1 week after noise-induced severe hearing loss in a mouse model (Basta et al., 2005).

In addition to these neuronal changes, neural projections may also be revised by the sensory signal input in animals undergoing development. Analyses of brainstem evoked potentials and the frequency of sensory evoked potentials in the auditory cortex of cats showed that signal input did not affect the process of axonal projection in the auditory brainstem area of prenatal cats (Kandler and Friauf, 1993). But if cochlear changes occur in the developmental stage in animals, the proportion of crossed and uncrossed fibers projecting from the cochlear nucleus to the inferior colliculus in adulthood may be different compared with animals showing normal development. More importantly, this only occurs in first three postnatal months; when cochlear changes in adulthood, animals will display minor forms of the aforementioned phenotypes, suggesting that early cochlear alteration during development may lead to more widespread plasticity in the projection of cochlear nerve fibers (Zhang et al., 2001). Moreover, researchers found that the neural projections from the dorsal nucleus of the lateral lemniscus to the inferior colliculus were affected, and the pattern of banded layers in the inferior colliculus was disrupted by both unilateral and bilateral cochlear resection (Franklin et al., 2008). Except for the ascending pathway, studies in bat revealed that descending corticofugal system was involved in experience-dependent plasticity and the best frequency (BF) shifts evoked by electrical stimulation (Gao and Suga, 2000).

Cochlear damage-induced sensory afferent blockade leads not only to different levels of apoptosis and degradation in the spiral ganglion but also to a noticeable change in synaptogenesis. Blockade of auditory afferent activity may result in the rearrangement of both neuronal and non-neuronal networks in the auditory brain stem. It may also alter cell morphology, cell network, and cell-cell relationships. Janz and Illing (2014) demonstrated that unilateral blockade of sensory afferent input to the cochlear nucleus significantly changed the morphology of microglial cells in the ventral cochlear nucleus (VCN). Through evaluation of molecular markers in different periods after cochlear ablation, adaptive changes of mitogen-activated protein kinase (MAPK) signal pathway in microglial cells were deemed to be associated with the blockade of incoming signal, in contrast to what was observed in astrocytes and neurons. Moreover, microglial cells may be involved in the synaptogenesis of the VCN in a particular time period. Researchers considered previous findings and proposed that microglial cells may be involved in the plasticity of synapses and affect the synaptic transmission in the hippocampus (Janz and Illing, 2014). Apart from the microglial cells, astrocytes in the central nervous system are also related to the reconstruction of synaptic connections (Fredrich et al., 2013). Anyway, the molecular basis still need to be further explored. Previous studies have indicated that these two cell types are involved in cell-mediated immunity in the nervous system (Ransohoff and Perry, 2009), in the removal of dead cells and in the regulation of the redistribution of nerve fibers (Graeber and Streit, 2010; Schafer et al., 2012). Recent studies have attached importance to the significant impact of non-neuronal cells on the reconstruction of auditory neural network.

## Aural Deprivation-Induced Changes in Synaptic Transmission

The number of excitatory neurons is more than that of inhibitory neurons, but inhibitory synapses still have a profound impact on auditory plasticity when deafness (Sanes and Kotak, 2011). Both excitatory and inhibitory synaptic properties in auditory cortex can be changed by aural deprivation. These changes may be sustained into adulthood and enhance the sensitivity of cortical neurons towards the remaining signal input (Kotak et al., 2005, 2008). Kotak studied pyramidal neurons in gerbils with hearing loss during development, and their results demonstrated that these neurons showed a depolarized resting membrane potential, increased input resistance, and increased rate of sustained discharge; in addition, the evoked excitatory synaptic responses of thalamocortical and intracortical neurons were significantly enhanced and became highly sensitive to an N-methyl-D-aspartate (NMDA) receptor antagonist. Following this electrophysiological study, researchers found that NMDAergic transmission was elevated by studying related indicators, such as amplitude and current conduction, and the evoked GABA release from cortical neurons in the sensorineural hearing loss group inhibited the synaptic response and significantly reduced the amplitude (Kotak et al., 2005). These results suggested that a central self-balancing mode exists, but the mechanism that interferes with the self-balancing mode after aural deprivation remains unclear.

Many studies have reported that aural deprivation can delay inhibitory synaptic neuronal development in the lateral superior olivary nucleus (LSO) in rats, thereby blocking inhibitory synapse formation and the associated maturation of the axonal and dendritic morphology (Kakazu et al., 1999; Hassfurth et al., 2009). In addition, the changes in chloride ions within the LSO neurons observed in the rats after aural deprivation confirmed the inhibitory synaptic transmission can be affected from another perspective (Shibata et al., 2004). The LSO can receive the excitatory projection of the ipsilateral cochlear nucleus and the inhibitory projection of the lateral trapezoid body. Hassfurth et al. (2009) studied the role of the hyperpolarization-activated inward current (*I*_h_), which could regulate neural excitability and improve the time analytical precision of the signal input from both ears. Through whole-cell patch-clamp analysis, the results demonstrated that cochlear resection prior to the development of hearing increased the *I*_h_ in the LSO and reduced the *I*_h_ in the medial nucleus of the trapezoid body (MNTB), resulting in the formation of a depolarized resting membrane potential in the LSO and decreasing the neuronal input resistance.

Besides, both GABAergic and glycinergic synaptic transmissions constitute inhibitory neurotransmission in the central nervous system, which may be closely related to plasticity changes in the auditory centers (Sarro et al., 2008). Studies have confirmed that early aural deprivation leads to a maturation disorder in GABAergic neurotransmission (Kotak et al., 2008; Takesian et al., 2010). However, the occurrence of conductive hearing loss in adulthood does not appear to result in changes in the amplitude and to decay timing of inhibitory currents (Takesian et al., 2012). Inferior colliculus (IC) is the key relaying nucleus, which has a complex reaction to acoustic stimulations. Because it accepts the innervation of both the ascending (from cochlea) and descending (from cortex) pathway, the plasticity of inhibitory synaptic transmission in IC seems remarkable recently. Vale’s study found that unilateral cochlear ablation would affect the inhibitory synapse of IC, and these changes mainly included postsynaptic modifications in the contralateral IC and presynaptic changes in the ipsilateral IC (Vale et al., 2004). Neural activity, neurotransmitters, receptors and inhibitory postsynaptic potential (IPSP) all can be modified owing to the aural deprivation. Reversing these plastic changes (e.g., changes in inhibitory levels in the auditory center) caused by auditory damage may be a new research theme in the future. In a recent study, GABA_A_ receptor α1 subunit-specific agonists and a selective GABA reuptake inhibitor (SGRI) were used to promote GABAergic neurotransmission and to restore the reduced strength of inhibitory postsynaptic currents caused by auditory damage. However, the GABA_B_ receptor agonist could not achieve this goal, suggesting the activation of GABA_A_ receptors may be necessary for promoting the maturation of central inhibitory transmission (Kotak et al., 2013).

In addition to quantifying altered levels of neurotransmitters, previous studies have investigated changes in the expression levels of NMDA and α-amino-3-hydroxy-5-methyl-4-isoxazolepropionic acid (AMPA) glutamate receptors following aural deprivation induced by amikacin treatment during development, and the result demonstrated that decreased mRNA expression of the NR1 (NMDA receptor 1), NR2a and NR2b subunits of NMDA receptors as well as the flop subunit of the AMAP receptor in the bilateral cochlear nucleus. However, the expression of GABA_A_ subunits (e.g., α1, β1, and γ2) as well as the flip subunit of the AMPA receptor was up-regulated (Marianowski et al., 2000). Subsequently, this team found that the intracochlear electrical stimulation on auditory pathways of these deaf neonatal mice could increase the expressions of NMDA receptor, AMPA receptor and GABA_A_ receptor in the cochlear nucleus and in the central nucleus of the inferior colliculus, so researchers suggested early electrical stimulation might be a way to maintain neuronal networks (Liao et al., 2000). Neurons with NR2b mRNA expressing distribute in different layers of the auditory cortex. Maybe due to the variance of input and output in these layers, the degree of the decreasing mRNA is not always consistent; and the NR2B mRNA of the layer 5 reduced by the maximum of 36.8% (Nordang et al., 2000). More and more research discovered that the NMDAR plays a crucial role in the experience-dependent synaptic plasticity (Sun et al., 2005). The subtype constitute of NMDAR, NR2A/NR2B, seems to change following the age-related development in the early postnatal days, and remarkably it can be regulated by aural deprivation (Liao et al., 2000; Sun et al., 2005). As a research proved, the early aural deprivation can decrease the expression levels of NR2B mRNA in auditory cortex and eliminate the transient peak of mRNA on postnatal day 21 without changing the distribution pattern of NR2B mRNA-positive neurons (Bi et al., 2006). The change of NMDAR can modify the presynaptic transmitter release and the excitatory postsynaptic currents (EPSCs), which affect the activity of neurons and reorganize the synaptic plasticity (Hsieh et al., 2002). For a single synapse, the long term potentiation (LTP), which is the basic mechanism of the learning and memory, may be mediated by the activation of NMDAR. By extracellular stimuli experiment at layer 6, Kotak found that layer 5 neurons more likely displayed long term depression (LTD) in sensorineural hearing loss (SNHL) group than that in control, and researchers further believed that the long-term synaptic plasticity depends on normal auditory experience (Kotak et al., 2007).

Regarding neurotransmitters, earlier researchers believed that cochlear damage would inevitably cause a series of complex changes, including the release, binding, and reuptake of glutamatergic, glycinergic, and GABA energy pathway neurotransmitters (Potashner et al., 1997; Suneja et al., 1998a,b). The auditory center (e.g., the inferior colliculus) could reduce the inhibitory effect through changes in tuning and discharging, thereby compensating for the defect in cochlear input activities (Salvi et al., 2000). Later studies examining changes in glutamic acid decarboxylase (GAD), which catalyzes GABA synthesis, suggested that decreasing in neurotransmitter levels (e.g., GABA) in the auditory center (e.g., the hypothalamus) may be the result of physiological compensation for weakened peripheral electrical activity (Pouyatos et al., 2004). Godfrey and colleagues reported numerous changes in neurotransmitters, receptors, and amino acids in the cochlear nucleus after cochlear resection. One of their recent studies revealed the impact of cochlear resection on the levels of amino acids (e.g., glutamate, GABA, glycine, aspartic acid, and serine) in the cochlear nucleus. Two days after cochlear resection, glutamate and aspartate levels were reduced, whereas levels of other amino acids were increased in the region of the cochlear nucleus that is largely dominated by the auditory nerve. Additionally, the level of GABA in the LSO decreased continuously, which may be associated with neuronal functions in the lateral olivocochlear system (Godfrey et al., 2014). Moreover, Godfrey and Jin studied the changes in M-like cholinergic receptor-binding materials and choline acetyl transferase after unilateral cochlear resection. They found that cholinergic neurons in the cochlear nucleus may be associated with the plasticity changes in the cochlear nucleus and the lateral superior olivary nucleus after cochlear damage. Changes in receptor-binding materials may reflect the plasticity in receptors lacking auditory nerve domination. This study provided a fundamental basis for the investigation of functional changes (e.g., deafness and hyperacusis) in the auditory center (Jin et al., 2005; Jin and Godfrey, 2006).

## Changes in Cortical Protein and Gene Expression Induced by Aural Deprivation

Multiple in-depth studies have been performed to elucidate the molecular mechanism underlying morphological and functional changes in the auditory center after aural deprivation. These changes include up- or down-regulation of proteins and genes and alterations of signaling molecules. Hutson et al. (2007) used ^3^H-leucine (heavy hydrogen leucine) as a tracer for monitoring during a 48-h period following unilateral conductive hearing loss and observed a significant down-regulation of protein synthesis in bilateral medial superior olive nuclei. However, the protein synthesis of bilateral medial superior olive nuclei and the ipsilateral trapezoid body was increased 6 h after the operation. Wang and Rubel (2008) studied the protein expression of microtubule associated protein 2 (MAP2) in the nucleus laminaris following unilateral cochlear resection, then they suggested that the protein distributed in the dendrites, perikarya, and postsynaptic density structures may play an important role in the dendritic structural modeling induced by deafferentation. After the deafferentation of cochlear nucleus neurons, both degeneration and regeneration could be observed. For example, a previous study showed that cochlear resection or noise damage might trigger the growth of nerve fibers and presynaptic terminals in the VCN. This regeneration largely appeared after degeneration and significantly at approximately 6–8 months after cochlear damage (Kim et al., 2004). Kraus et al. (2009) applied carboplatin to induce cochlear hair cell loss in the oval window of chipmunks and confirmed the expression of a growth-associated protein (i.e., GAP-43). The results showed that after 15 and 31 days of carboplatin treatment, up-regulated expression of GAP-43 occurred in the carboplatin-treated ipsilateral VCN, whereas little change in GAP-43 expression was observed in the dorsal cochlear nucleus. In the VCN, the up-regulation of GAP-43 expression in the audio high frequency area was greater than in the audio low frequency area. This result corresponded to the gradient-like degeneration pattern observed from the base-apex turn of cochlear outer hair cells. In addition to growth- and degeneration-related proteins, calcium-binding proteins (CaBPs) are also affected following unilateral cochlear resection in the early developmental stage (Hatano et al., 2009). This type of protein is considered to be associated with neurotransmitter release, ion channel functions of the neuronal membrane, and intracellular activities.

Except the reported protein changes, some researchers have compared the genetic changes in the primary auditory cortex of rats before and after an operation to induce bilateral cochlear damage. The results showed that the expression of early growth response 1 through 4 (*Egr1–4*) and *c-fos*, among the immediate early genes, and activity-regulated cytoskeleton associated protein (*Arc*), synaptogyrin 1 gene (*Syngr 1*), and brain-derived neurotropic factor (*Bdnf*), among neural plasticity genes, in the aural deprivation group was first reduced and then increased from the 2nd to the 4th week after the operation. The expression of the gamma-aminobutyric acid A receptor, alpha 5 (*Gabra5*), cholinergic receptor nicotinic beta polypeptide 3 (*Chrnb3*), and cholinergic receptor nicotinic epsilon (*Chrne*) genes, which are associated with neurotransmission, was reduced in the 12th week after the operation (Oh et al., 2007). Moreover, a study by Suneja and Potashner (2003) addressing relevant kinase and signaling molecules in cerebral nuclei following unilateral cochlear resection showed that the extracellular-signal-regulated kinase (ERK) pathway may be an important pathway for regulating neuronal signal transduction after the blockade of auditory input. This enzymatic activity may affect gene expression and the mechanism of cytoplasmic regulation. It may also be one of the molecular mechanisms involved in the plasticity changes observed following unilateral cochlear resection.

## Process of Cross-Modal Reorganization

As we know, humans obtain most of the external information through vision and hearing. Each modality can partly compensate for a defect of the other modality under certain conditions (Bulkin and Groh, 2006). For example, visual information can improve the speech perception of humans in noisy environments (Bishop and Miller, 2009), while salient sound can improve perceptual process of a subsequent visual target that locate in close spatial proximity (McDonald et al., 2013). Cross-modal reorganization has been investigated in numerous studies of vision or auditory deprived animals. For instance, totally blind individuals perform better in sound localization than sighted individuals or others still with residual peripheral vision (Lessard et al., 1998), also people blinded at an early age may have a better perception of chords and become a better sense in the direction of pitch change (Gougoux et al., 2004). Another type of change involves processing of visual input signals following aural deprivation to compensate for the absent tuning capability and sound location, thereby activating part of the auditory cortex through a visual input (Lomber et al., 2010; Meredith and Allman, 2012). The peripheral vision and motion-processing capabilities of congenitally hearing-impaired cats were shown to be enhanced to recruit the higher-order auditory cortex to improve their performance in some tests (Meredith et al., 2011). A similar phenomenon occurs in humans. For example, some regions of the auditory cortex were shown to be activated in patients with congenital or postlingual deafness when processing the visual motion or changing complex image (Vachon et al., 2013). To study the extent of aural deprivation that causes cross-modal cortical reorganization, Lambertz et al. (2005) designed a controlled trial to assess the results through fMRI, in which patients with different extents of aural deprivation watched a video with markup language and black bars alternately; and they ultimately found patients with complete hearing loss showed an activation of the primary auditory cortex when they processed the markup language, whereas patients with residual hearing did not in the same test. A recent study by Campbell and Sharma (2014) applied electroencephalography to record and assess the visually evoked potential in patients with hearing loss and to further compare their results with a control group. Patients with hearing loss exhibited a large P1, N1, and P2 amplitude with a shortened N1 latency. A positive component P2’ was observed after an abnormal P2. The visual cross-modal reorganization of these patients may begin in the early stage of hearing loss and probably be an important factor in determining the prognosis of patients with hearing loss.

The mechanism underlying the cross-modal reorganization becomes a hot area these years. A study revealed that the enhanced inhibitory conduction reduced the effect produced by an abnormal visual signal input and suggested that, following cross-modal reorganization, the plastic change in inhibition may play a role in the reorganization of the sensory cortex and pharmacological treatment just like blocking GABA_A_ could be possible for patients who have sensory deprivation (Mao and Pallas, 2013). Furthermore, according to a recent study, when visual deprivation, thalamocortical synapses were potentiated in primary auditory cortex (A_1_), but not in primary visual cortex (V_1_); and the cross modal TC-plasticity was effectively recruited in V1 when aural deprivation in adult mice. According to the result, researchers suggested that multimodal training paradigms may benefit individuals with auditory processing disorders (Petrus et al., 2014). Except for anatomical and functional reorganization of cortical circuits, at the cell and molecular level, the cross-modal plasticity is also related to changes in the constitution of synaptic receptors, such as the AMPA receptor subunit (Goel et al., 2006).

## Clinical Problems Induced by Plastic Changes in the Auditory Cortex Following Aural Deprivation

Most studies on aural deprivation were conducted in animal models. In recent years, positron emission tomography-computed tomography (PET-CT), functional magnetic resonance imaging (fMRI), and evoked potentials have been widely used to allow imaging, becoming important techniques for studying development and determining plasticity changes in the auditory center. Through the detection of central metabolism and electrical activities, these methods allow non-invasive imaging analyses of patients’ brains. A previous study demonstrated that the glucose metabolism of the local cerebral cortex of humans less than 16 years of age first increases and then declines as a positive waveform. Glucose metabolism reaches its peak at 6 years old. This change is consistent with the timing of cortical synaptic maturation during development (Chugani, 1998). Hsu et al. (2009) applied PET-CT for studying the auditory cortex in a rat model to assess changes in glucose metabolism in the rat auditory center in a non-invasive manner. The result showed that unilateral cochlear resection reduced the radioactivity of the lateral inferior colliculus and the auditory cortex area. Another research group employed fluorodeoxyglucose (FDG)-PET to detect and evaluate five adult male cats regarding the metabolic activities of their whole cortex and vertically compared images corresponding to normal hearing cats and hearing impairment cats after 4, 9, 24, and 33 months. The result showed that metabolic decay was most significant in the primary auditory cortex and the temporal lobe area of animals with bilateral hearing impairment in the 9th month. This decay was less dramatic in the 24th month and disappeared in the 33rd month. Moreover, the metabolism of bilateral occipital regions (including the primary visual cortex of the occipital region and thalamus) was accelerated in the 33rd month, suggesting the existence of compensatory activity in the visual cortex after hearing impairment (Park et al., 2010). With such innovations in equipment and research methods, imaging studies for the functional assessment of sensory organs will become more abundant, further advancing the study of changes in neural activity in the cortical region following aural deprivation.

In clinical practice, researchers have demonstrated that the duration of aural deprivation affects functional recovery after interventions in hearing-impaired individuals. Patients with binaural hearing impairment who only wore one hearing aid showed a worse outcome of unaided ear than those wearing no hearing aids in both ears (Schnupp and Carr, 2009). To a certain extent, the binaural alternate use of hearing aid in children with bilateral symmetrical hearing loss may prevent the auditory plasticity caused by aural deprivation from occurring. No significant decline in speech recognition was observed during the early application of hearing aids (Hattori, 1993).

Factors affecting the efficiency of hearing aid have been widely discussed in the field of clinical auditory interventions. At first, during the critical period of auditory development, environmental sound stimulation is very important in the development of the auditory cortex. Electrophysiological examinations, hearing tests, and behavioral audiometry analyses were performed to screen for hearing impairment in newborns and 9-month-old infants to allow 3–5 years of rehabilitation training to be offered. Following the rehabilitation training, the children who were screened for hearing impairment in the neonatal stage displayed better development, communication, activity, and other indicators of recovery than the children who were screened for hearing impairment at 9 months of age (Korver et al., 2010). Another study had compared recovery between hearing-impaired children who received an auditory intervention before 3.5 years and after 7 years. The results demonstrated that hearing-impaired children who received auditory intervention early exhibited a rapid recovery in their auditory evoked potentials (Sharma et al., 2005). Kral has suggested that the sensitive period for human auditory cortex may be before 6.5–7.0 years of age and that the younger the patients who receive a cochlear implantation (in the first 3.5–4.0 years of life, and best before 2 years old), the stronger that their neural plasticity will be (Kral and Sharma, 2012). Thus, early screening and promptly treatment involving hearing aid intervention are essential to improve the long-term prognosis of hearing-impaired children.

In addition, a prognostic study on patients who received cochlear implantation showed that patients with a low preoperative temporal cortex metabolism may display better speech recognition and implantation integrity than patients with normal metabolism. These differences may be due to the presence of cross-modal reorganization. Prolonged aural deprivation results in reorganization of corresponding regions in the auditory cortex as well as increased neural activity and an elevated metabolic rate (Lee et al., 2003b, 2007). A study by Lee et al. (2001) also showed that the lower impact of plasticity mechanisms on cross-modal reorganization, the better the auditory recovery of the patients will be. To summarize the previous findings, we cannot ignore that early interventions mean a lot to hearing-impaired children, and any kinds of non-acoustic activities prior to the auditory intervention such as the use of sign language, lip reading, and other visually stimulating signals should be avoided. Generally speaking, in children undergoing rehabilitation training after the implantation of hearing devices, the cross-modal reorganization mechanism that disturbs the recovery of the auditory center should be avoided.

Recently, researchers found some detection methods which could be used to evaluate the effect of auditory intervention. A study used the median visual evoked potential and the median nerve somatosensory evoked potential (SEP N20) to predict the prognosis of hearing-impaired children before the cochlear implantation. The results demonstrated that children in whom cochlear implantation was delayed and initially relied on sign language communication displayed overexpression of SEP in the region of the left temporal cortex, suggesting the presence of cross-modal reorganization (Charroó-Ruíz et al., 2013). Beyond that, Alfelasi et al. (2013) proposed that a named transtympanic promontory stimulation test (TPST) may be another good strategy for predicting therapeutic efficacy.

## Future Direction

Similar to other sensory modalities, the peripheral hearing input is essential for the development and functional maturation of the auditory center. Long-term blockade of peripheral signal or an asymmetric input throughout life may induce structural and functional reorganization of the auditory center. These effects may be due to changes in a large number of molecules, proteins, genes, and signaling pathways. During the critical period of auditory development, the auditory experience, environmental experience, and different damaging factors may directly or indirectly cause different degrees of central adaptive changes. Some changes may remain to adulthood in individuals and affect their auditory skill acquisition. In the future, research of aural deprivation-induced plastic changes may continuously increase at different levels in the aforementioned areas. Molecular mechanisms corresponding to the observed morphological changes, correlations between different functional changes, the synaptogenesis, the degeneration of nerve fibers, the reconstruction of neural circuits, neurotransmitter regulation and the activation of many proteinases will become hot research topics. With the increasingly attention of translational medicine, research findings involving aural deprivation and central plasticity may affect clinical auditory rehabilitation strategies. That reversing the reorganization of the auditory center caused by aural deprivation will allow patients with hearing impairment to benefit from early interventions and proper auditory rehabilitation trainings, although this is still a challenge for both neuroscientists and clinical audiologists.

## Conflict of Interest Statement

The authors declare that the research was conducted in the absence of any commercial or financial relationships that could be construed as a potential conflict of interest.
